# Introgression of Chromosome 3Ns from *Psathyrostachys huashanica* into Wheat Specifying Resistance to Stripe Rust

**DOI:** 10.1371/journal.pone.0021802

**Published:** 2011-07-08

**Authors:** Houyang Kang, Yi Wang, George Fedak, Wenguang Cao, Haiqin Zhang, Xing Fan, Lina Sha, Lili Xu, Youliang Zheng, Yonghong Zhou

**Affiliations:** 1 Triticeae Research Institute, Sichuan Agricultural University, Chengdu, Sichuan, China; 2 Eastern Cereal and Oilseed Research Centre, Department of Agriculture and Agriculture-Food Canada, Ottawa, Ontario, Canada; 3 Key Laboratory of Crop Genetic Resources and Improvement, Ministry of Education, Sichuan Agricultural University, Yaan, Sichuan, China; East Carolina University, United States of America

## Abstract

Wheat stripe rust is a destructive disease in the cool and humid wheat-growing areas of the world. Finding diverse sources of stripe rust resistance is critical for increasing genetic diversity of resistance for wheat breeding programs. Stripe rust resistance was identified in the alien species *Psathyrostachys huashanica*, and a wheat- *P. huashanica* amphiploid line (PHW-SA) with stripe rust resistance was reported previously. In this study, a *P. huashanica* 3Ns monosomic addition line (PW11) with superior resistance to stripe rust was developed, which was derived from the cross between PHW-SA and wheat J-11. We evaluated the alien introgressions PW11-2, PW11-5 and PW11-8 which were derived from line PW11 for reaction to new *Pst* race CYR32, and used molecular and cytogenetic tools to characterize these lines. The introgressions were remarkably resistant to CYR32, suggesting that the resistance to stripe rust of the introgressions thus was controlled by gene(s) located on *P. huashanica* chromosome 3Ns. All derived lines were cytologically stable in term of meiotic chromosome behavior. Two 3Ns chromosomes of *P. huashanica* were detected in the disomic addition line PW11-2. Chromosomes 1B of substitution line PW11-5 had been replaced by a pair of *P. huashanica* 3Ns chromosomes. In PW11-8, a small terminal segment from *P. huashanica* chromosome arm 3NsS was translocated to the terminal region of wheat chromosomes 3BL. Thus, this translocated chromosome is designated T3BL-3NsS. These conclusions were further confirmed by SSR analyses. Two 3Ns-specific markers *Xgwm181* and *Xgwm161* will be useful to rapidly identify and trace the translocated fragments. These introgressions, which had significant characteristics of resistance to stripe rust, could be utilized as novel germplasms for wheat breeding.

## Introduction

Stripe rust, caused by *Puccinia striiformis* Westend. f. sp. *tritici* Eriks. (*Pst*), is one of the most important diseases in common wheat production worldwide, especially in the cooler and wetter environments [1]. It is the most destructive disease in autumn-sown wheat in northwestern and southwestern China, where stripe rust resistance is a major breeding objective [2]. Fifteen countrywide stripe rust epidemics have been recorded since 1950, and losses of 6.0, 3.2, 1.8 and 1.3 million metric tons of wheat occurred during 1950, 1964, 1990 and 2002, respectively [3]. Since the appearance of new *Pst* race CYR32, almost all of the resistance genes used in wheat breeding program of southwestern China (*Yr1*, *2*, *3*, *4*, *6*, *7*, *8*, *9*, *17*, *18*, *20*, *21*, *22*, *25*, *27*) have become susceptible, resulting in the 2002 epidemic [3], [4]. It is, therefore, urgent to identify new stripe rust resistance genes and to use more effective genes in wheat breeding programs. The wild relatives of wheat are important sources of resistance genes to many diseases and pests including stripe rust and, to date, more than 10 stripe rust resistance genes have been transferred to durum or common wheat from various wild relatives [5], [6]. Some of these new alienderived genes are effective against CYR32 [7].

*Psathyrostachys* Nevski (Ns genome), a small genus of the tribe Triticeae, has provided a number of desirable genes, such as those for resistance to biotic and abiotic stresses [8]. Therefore, the *Psathyrostachys* Ns genome can be used as donor to provide genes for the genetic improvement of wheat crops. Wide crosses between *Psathyrostachys* and common wheat began in the 1980s and were first reported in 1988 [9]. Since then, the hybridization of common wheat with *Psathyrostachys* species such as *P. juncea*, *P. fragilis* and *P. huashanica* has shown considerable progress [9]–[11]. *Psathyrostachys huashanica* Keng ex Kuo (2n = 14, NsNs), a perennial cross-pollinating plant, is distributed in Huashan Pass in the Qinling Mountains of Shaanxi Province in China [8], [12]. It is an endemic species in China that has been listed as an endangered and imperatively protected wild species (Chinese National Forest Bureau and Agriculture Ministry 1999). *P. huashanica* is characterized by a dwarfed stature, early maturity, with resistance to drought, salinity, wheat stripe rust and take-all fungus. It is therefore a potentially useful germplasm source for wheat improvement [11], [13]–[15]. In order to transfer desirable traits from *P. huashanica* into wheat, intergeneric hybrids of common wheat with *P. huashanica* had been obtained [11], [16]. Progeny lines with single *P. huashanica* chromosomes incorporated into the wheat genome, carrying resistance to wheat take-all fungus disease, had been obtained either as chromosome additions or substitutions. However, the homoeologous relationship of the added *P. huashanica* chromosomes in these lines had not been determined [17]–[19]. Zhao et al. developed and identified a wheat-*P. huashanica* addition line (H9021-28-5) carrying HMW-GS, LMW-GS and gliadin genes of *P. huashanica* [20].

In April 2004, our research team successfully hybridized common wheat and *P. huashanica* without using embryo rescue [16]. By chromosome doubling of F_1_ hybrids with colchicine treatment, a new intergeneric amphiploid (PHW-SA, 2n = 8x = 56, AABBDDNsNs) between common wheat and *P. huashanica* was synthesized [15]. Kang et al. characterized the amphiploid using molecular cytological tools, and reported that the stripe rust resistance of the *P. huashanica* parent was completely expressed [21]. The production of the amphiploid serves not only to maintain the germplasm, but also provides an effective and rapid way of introgressing desirable traits from related species into cultivated wheat [22]. Therefore, the production of wheat- *P. huashanica* introgression lines, which had transferred the chromatin carrying the stripe rust resistance gene into wheat background, may provide new sources of resistance and eventually resistant cultivars.

The objectives of this study were (1) to develop the new introgression lines with stripe rust resistance; (2) to determine the genetic control of resistance to stripe rust of these lines; and (3) to characterize the chromosome constitution of those lines by genomic in situ hybridization (GISH), Giemsa C-banding, simple sequence repeats (SSR) and meiosis analyses.

## Results

### Development of the wheat-*P. huashanica* introgression lines

**Figure 1:**
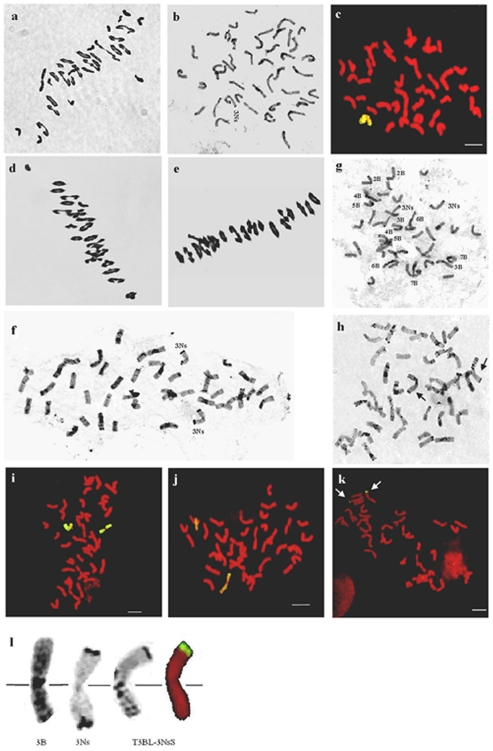
Meiotic pairing configuration, C-banding and GISH patterns of wheat- *P. huashanica* progeny lines. **a** Meiotic metaphase I pairing in the PHW-SA×J-11 F_1_ hybrid: 2n = 49 with 13 I+15 II (ring)+3 II (rod); **b**, **c** C-banding and GISH patterns of PW11 (2n = 43), which contained 42 wheat chromosomes and one 3Ns chromosome of *P. huashanica* (yellow-greenish); **d**, **e** Meiotic metaphase I cell of PW11-2 (2n = 44) and PW11-8 (2n = 42) with 2 I+18 II (ring)+3 II (rod) and 20 II (ring)+1 II (rod), respectively; **f** C-banding pattern of PW11-2 (2n = 44), which included 42 wheat chromosomes and a pair of 3Ns chromosomes of *P. huashanica*; **g** C-banding pattern of PW11-5 (2n = 42), where two wheat 1B chromosomes were replaced by a pair of 3Ns chromosomes of *P. huashanica*; **h** C-banding pattern of PW11-8 (2n = 42), which contained 40 wheat chromosomes, one pair of wheat- *P. huashanica* terminally translocated chromosomes (arrows indicate a pair of 3BL-3NsS translocated chromosomes); **i**–**k** GISH using *P. huashanica* total genomic DNA labeled with digoxigenin-11- dUTP as probe and Chinese Spring genomic DNA as block (The *P. huashanica* chromatin fluoresced yellow-greenish, and the wheat chromatin fluoresced red). **i** GISH pattern of PW11-2 (2n = 44); **j** GISH pattern of PW11-5 (2n = 42); **k** GISH pattern of PW11-8 (2n = 42) (arrows indicate 3BL-3NsS translocated chromosomes); **l** Magnified picture of the translocated chromosome, from left to right: C-banded wheat chromosome 3B, C-banding of *P. huashanica* chromosome 3Ns, C-banding and GISH image of the 3BL-3NsS translocated chromosome.

The wheat landrace J-11 was crossed with the amphiploid PHW-SA as the pollen parent, and the F_1_ hybrids were obtained successfully. The chromosome configuration of the F_1_ hybrids (2n = 49) averaged 8.05 univalents, 16.91 ring bivalents, 3.52 rod bivalents and 0.03 trivalents per PMC (Figure 1a). The F_1_ hybrids were backcrossed with J-11, then seeds selected from the BC_1_F_1_ plants were bulked and advanced to the BC_1_F_2_ generation. Twelve lines were selected and evaluated for stripe rust reaction in the greenhouse. Further evaluation of stripe rust reaction with point inoculation indicated that the line PW11 had high levels of resistance. C-banding and GISH data indicated that PW11 was a monosomic addition line containing one 3Ns chromosome of *P. huashanica* (Figure 1b, c). In the selfed progenies of the line PW11, the frequency of plants with the added *P. huashanica* 3Ns chromosome was about 36.4%, indicating that the monosomic addition line can be repeatedly selected and used as a continuous source for transferring *P. huashanica* chromatin into wheat. This line was selected and used for chromosome characterization and stripe rust evaluation. Following two to four generations of selfing, accompanied by cytogenetic analysis, lines PW11-2, PW11-5 and PW11-8 with 2n = 42 or 44 were developed from line PW11. Thirteen plants derived from line PW11 with different chromosome numbers of 2n = 42, 43 or 44 were used to examine the association of stripe rust resistance with the alien chromosome.

### Stripe rust reaction of wheat–*P. huashanica* introgression lines

**Figure 2:**
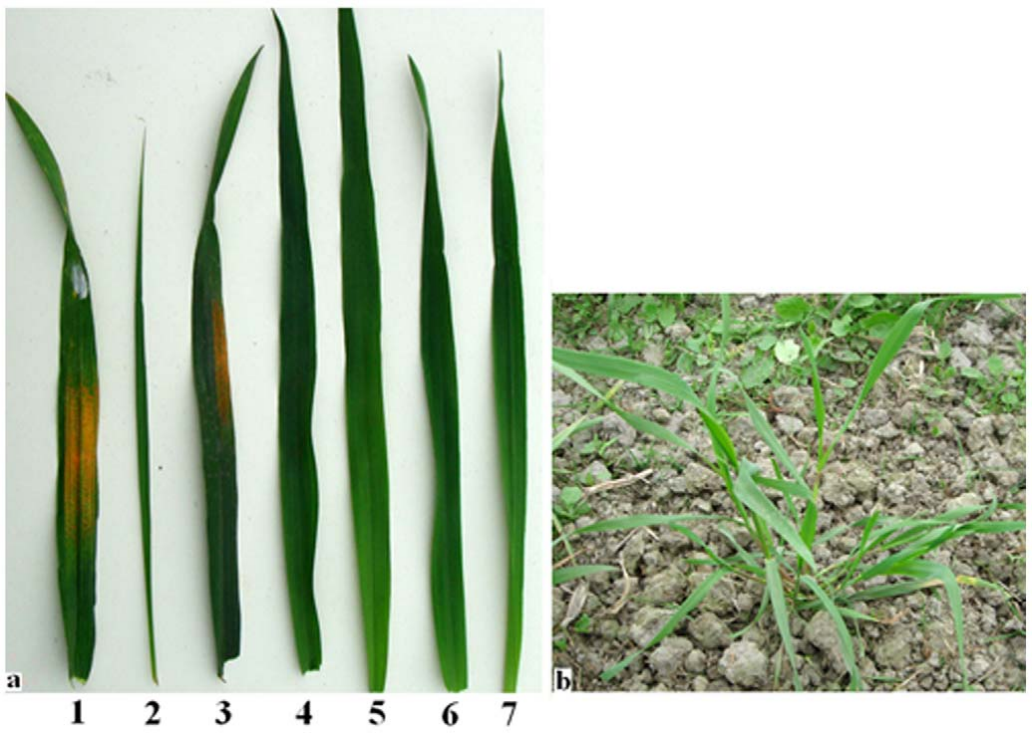
The symptom after four weeks by *Puccinia striiformis tritici* (*Pst*) race CYR32 inoculation. **a** leaf symptom, ***1*** Mingxian169, ***2*** *P. huashanica*, ***3*** J-11, ***4*** PHW-SA, ***5*** PW11-2, ***6*** PW11-5 and ***7*** PW11-8; **b** the plant symptom of PW11-8.

*P. huashanica*, J-11, PHW-SA, Mingxian169, PW11-2, PW11-5 and PW11-8 were evaluated with *Pst* race CYR32 at Chengdu, Sichuan, China. J-11 and Mingxian169 were susceptible to the race, showing infection of types 3 and 4, respectively. PHW-SA, *P. huashanica*, PW11-2, PW11-5 and PW11-8 were resistant to CYR32, showing only 0 or 0; infection types (Figure 2). This indicates that the stripe rust resistance gene(s) derived from *P. huashanica* accession ZY3157 is completely expressed in the wheat–*P. huashanica* introgression lines PW11-2, PW11-5 and PW11-8.

### Meiotic analysis

The meiotic behavior (metaphase I pairing) of introgression lines was quite regular with very low frequencies of both multivalent configurations and unpaired chromosomes. At meiotic metaphase I, the average frequency of univalent chromosomes ranged from 0.30 to 1.02 per cell, as shown in Table 1. The maximum number of univalents observed was 4 per cell. In the three lines, the majority of these lacked univalents. An average of 21.49 bivalents were observed, and 1.02 univalents per cell occurred in the line PW11-2 with *2n* = 44 (Figure 1d). The line PW11-5 with a chromosome number of 42 had an average of 20.72 bivalents and 0.56 univalents. The chromosome configuration of the PW11-8 plants (*2n* = 42) was 0.30 univalents, 19.54 ring bivalents and 1.31 rod bivalents, per PMC (Figure 1e). The low incidence of univalents and multivalents confirmed that these lines were basically cytologically stable.

**Table 1:** Means of chromosome pairing frequency at meiotic metaphase I in wheat- *P. huashanica* introgression lines.

Line	Chromosome No.	No. of cells	Chromosome configuration						Chiasma per cell
			Univalent	Bivalent			Trivalent	Quadrivalent	
				rod	Ring	Total			
PHW-SA	56	60	1.15 (0–8)	5.25 (0–16)	22.09 (12–28)	27.34 (24–28)	0.03 (0–1)	0.02 (0–1)	49.55 (40–56)
PW11-2	44	100	1.02 (0–4)	2.81 (1–7)	18.68 (14–21)	21.49 (20–22)	–	–	40.17 (35–43)
PW11-5	42	98	0.56 (0–4)	1.92 (0–6)	18.80 (15–21)	20.72 (20–21)	–	–	39.52 (35–42)
PW11-8	42	114	0.30 (0–2)	1.31 (0–4)	19.54 (17–21)	20.85 (20–21)	–	–	40.39 (37–42)

### C-banding analysis

C-banding of mitotic metaphase cell of introgressions allowed identification and distinction *P. huashanica* chromatin from wheat chromosomes. Based on similarities with the standard C-banded karyotype of common wheat and *P. huashanica* [23], [24], all 42 wheat chromosomes were identified in PW11-2 (2n = 44), plus the two added *P. huashanica* 3Ns chromosome (Figure 1f). Similarly, a pair of *P. huashanica* 3Ns chromosomes were also observed in line PW11-5 (2n = 42) (Figure 1g), which replaced a pair of wheat 1B chromosomes. Two wheat 3B long-arm chromosomes with a sharp telomeric band from *P. huashanica* 3Ns short-arm chromosome at the terminal region were found in line PW11-8 (2n = 42) (Figure 1h).

### GISH detection

Standard GISH analysis, employing wheat CS DNA for blocking and digoxigenated total genomic DNA of *P. huashanica* as a probe was used to analyze the genomic constitution of the three derived introgression lines. The lines PW11-2 (*2n* = 44) and PW11-5 (2n = 42) were shown to have a pair of *P. huashanica* chromosomes (Figure 1i, j). The line of PW11-8 (*2n* = 42) was found to have one pair of terminally translocated chromosomes (Figure 1k). They were likely homozygous chromosomes based on meiotic investigation. C-banding and GISH of root tip chromosomes confirmed that the lines PW11-2, PW11-5 and PW11-8 were wheat-*P. huashanica* 3Ns chromosome addition line, 3Ns/1B substitution line and T3BL-3NsS small-segment-translocation line (SS translocation) (Figure 1l), respectively.

**Figure 3:**
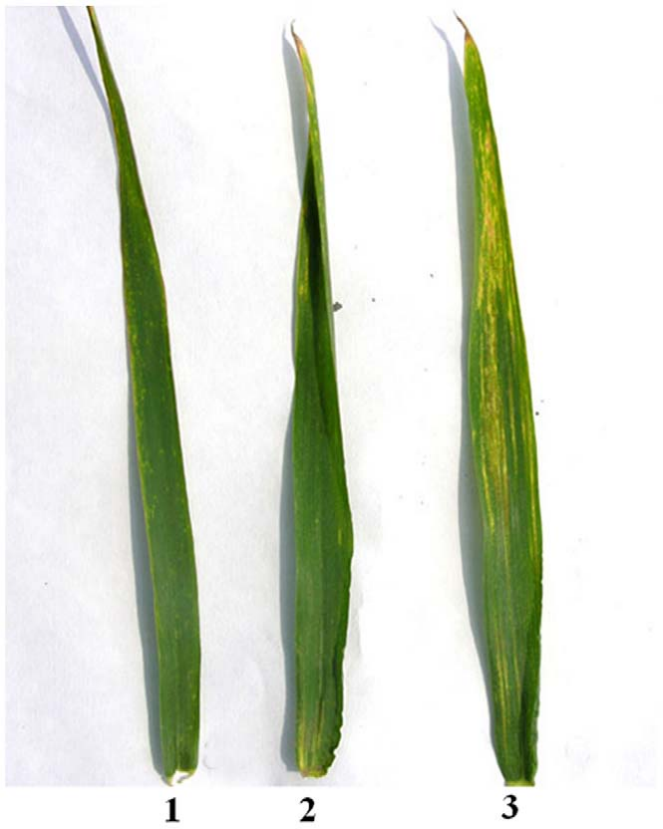
The leaf symptom after four weeks by *Puccinia striiformis tritici* (*Pst*) race CYR32 inoculation. ***1*** PW11, ***2*** the derivative line of PW11 (resistance to stripe rust) ***3*** the derivative line of PW11 (susceptible to stripe rust).

Thirteen plants derived from line PW11, which were different in resistance to stripe rust disease, were analyzed by GISH to confirm the association of *P. huashanica* chromosome with the resistance to stripe rust. Nine plants with more remarkably resistance to stripe rust carried one or two *P. huashanica* 3Ns chromosomes. Other plants without *P. huashanica* chromosomes did not differ from wheat cultivar J-11 for resistance to stripe rust (Figure 3). These results demonstrated an association between the *P. huashanica* 3Ns chromosome and the increased abilities of resistance to stripe rust in the wheat-*P. huashanica* progeny lines.

### SSR identification

**Figure 4:**
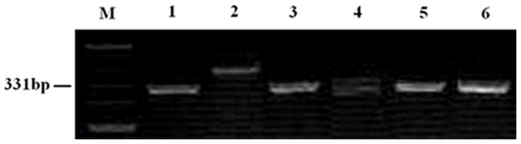
Agarose gel electrophoresis of the SSR marker *Xgwm161*, indicatiing the presence of the specific Ns fragment derived from *P. huashanica* in amphiploid PHW-SA, the wheat- *P. huashanica* introgression lines PW11-2, PW11-5 and PW11-8. *lanes*: ***M*** size standard, ***1*** *P. huashanica*, ***2*** J-11, ***3*** PHW-SA, ***4*** chromosome addition line PW11-2, ***5*** chromosome substitution line PW11-5, ***6*** chromosome translocation line PW11-8.

**Figure 5:**
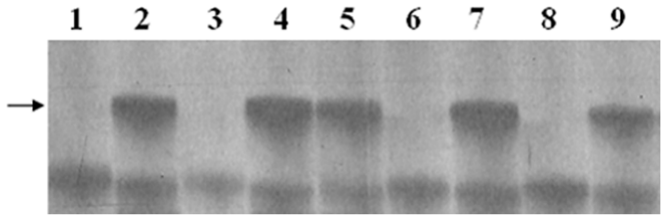
PCR amplification pattern that was produced using wheat SSR primer *Xgwm33* on chromosome 1B. The arrow indicates the band that was absent in the substitution line PW11-5. *lanes*: ***1*** *P. huashanica*, ***2*** J-11, ***3*** PW11-5, ***4*** PHW-SA, ***5*** PW11-2, ***6*** N1BT1A, ***7*** N1AT1B, ***8*** N1BT1D, ***9*** N1DT1B.

Thirty-one SSR primer pairs, which were polymorphic between J-11 and *P. huashanica* (ZY3157) and having known locations on different wheat chromosomes, were obtained through the screening of the 183 SSR primer pairs. These SSR markers were used to analyze the wheat- *P. huashanica* lines PW11-2, PW11-5 and PW11-8. *Xgwm161*, which maps proximal to the centromere of chromosome 3D, generated a polymorphic band between J-11 and *P. huashanica*, the amphiploid PHW-SA, PW11-2, PW11-5 and PW11-8 (Figure 4). This indicated that all the lines could carry group 3 chromatin from *P. hushanica*. Therefore, amplification products of this primer pairs could be used to develop the specific marker and used to detect *P. huashanica* chromatin in wheat-*P. huashanica* progenies. The diagnostic bands of the SSR markers, such as *Xgwm33* and *Xwmc419* on chromosome 1B, were missing in substitution line PW11-5, CS nulli-tetrasomic (NT) lines N1BT1A and N1BT1D (Figure 5), but all SSR markers on other chromosomes were present in PW11-5. This suggested that chromosome 1B of line PW11-5 had been substituted by a pair of *P. huashanica* chromosomes. The SSR markers on all wheat chromosomes amplified similar products in chromosome addition line PW11-2 and J-11 (data not shown), indicating the presence of all the wheat chromosomes.

**Figure 6:**
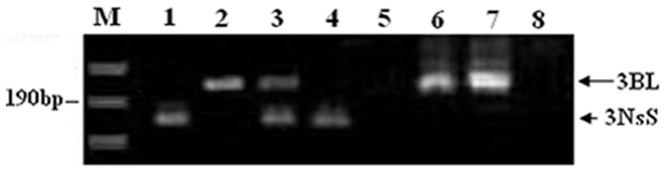
PCR amplification pattern that was produced using wheat SSR primer *Xgwm181* on chromosome 3B. The *long arrow* indicates the product associated with chromosome arm 3BL and the *short arrow* indicates the product from 3NsS, which indicated that the native J-11 chromosome 3BL fragment was replaced by the *P. huashanica* chromosome 3NsS fragment in the translocation line PW11-8. *lanes*: ***M*** size standard, ***1*** *P. huashanica*, ***2*** J-11, ***3*** PHW-SA, ***4*** PW11-8, ***5*** N3BT3A, ***6*** N3DT3B, ***7*** N3AT3B, ***8*** N3BT3D.

To determine whether the translocated chromosome segment involved wheat chromosome 3B, 22 SSR markers that were genetically mapped on wheat chromosome 3B were used to analyze the polymorphism on *P. huashanica*, J-11, PHW-SA, PW11-8, NT lines N3AT3B, N3BT3A, N3BT3D and N3DT3B. *Xgwm181*, which maps distal to the 3BL7-0.63-1.00 deletion breakpoint, amplified a fragment from the 3NsS and a different fragment from 3BL (Figure 6), indicating that a fragment derived from J-11 was replaced by a fragment of the *P. huashanica* donor in PW11-8. Therefore, amplification products of the the primer pairs can be used to detect *P. huashanica* chromosome 3NsS segment on a wheat chromosome.

## Discussion

### Isolation of the stripe rust-resistant wheat- *P. huashanica* introgression lines

Although genetic diversity in wheat was reduced during domestication, some of it can be restored through introgression from its progenitors or from the more distant wild relatives [25]–[27]. The conventional chromosomal manipulation by crossing between common wheat and the amphiploid has been widely utilized to transfer the alien genes to wheat. The amphiploids between wheat and species from genera *Secale*, *Thinopyrum*, *Agropyron*, *Dasypyrum*, and *Leymus* have been synthesized to produce many genotypes in wheat breeding [22], [28]–[30].

The superior resistance to stripe rust is useful in improving yield of wheat and can be achieved by the introgression of chromosomes or segments from the wild species related to wheat. *P. huashanica* has many useful traits such as abiotic-stress tolerances and disease resistances for wheat improvement, so a project aimed at transferring *P. huashanica* traits into wheat was initiated by crossing wheat cultivar J-11 with *P. huashanica* accession ZY3157 in 2004, which resulted in a number of progeny lines [16]. Among them, the amphiploid PHW-SA exhibited complete resistance to stripe rust in repeated evaluation and field observations [15]. In the present study, the line PW11 (2n = 43), which had high levels of resistance to stripe rust, was selected from PHW-SA/J-11//J-11 BC_1_F_2_ generation. The progeny lines PW11-2, PW11-5 and PW11-8 derived from the line PW11 were identified as having a *P. huashanica* 3Ns chromosome addition, 3Ns/1B substitution and 3BL-3NsS SS translocation lines by using *P. huashanica* genomic DNA as a probe in the presence of CS wheat genomic DNA as a blocker. The association of the *P. huashanica* chromosome with resistance to stripe rust was confirmed via analyzing line PW11 progeny plants segregating for the *P. huashanica* chromosome. By means of C-banding, GISH, and SSR analyses, the chromosome of *P. huashanica* in lines PW11-2, PW11-5 and PW11-8, which is responsible for resistance to stripe rust, was identified and designated as 3Ns. This is the first report on the transfer and genetic control of *P. huashanica* chromatin that enhances the ability of stripe rust resistance. Previously, Fu et al. reported two chromosome addition and substitution lines that were derived from common wheat cv. “7182” and *P. huashanica* that were resistance to wheat take-all fungus [17]. Zhao et al. developed and charactered the wheat-*P. huashanica* 5Ns/5A, 4Ns/3D substitution lines and 5Ns, 6Ns addition lines [18], [19]. However, these authors did not describe the resistance to stripe rust of the wheat- *P. huashanica* lines, and they failed to produce any chromosome translocation lines from the wheat- *P. huashanica* cross. In the present study, wheat-*P. huashanica* chromosome addition, substitution and translocation lines were developed and described. These lines have desirable resistant disease traits that were useful for wheat improvement.

### Identification of the wheat–*P. huashanica* introgression lines

Using C-banding techniques chromosome identifications are fast, reliable and economical [22], [31]. Wang et al. provided a C-banding karyotype of *P. huashanica*, and revealed that all the chromosomes exhibited strong bands only in their terminal regions [24]. Kang et al. reported that all *P. huashanica* chromosomes were completely discernible by C-banding in the wheat- *P. huashanica* amphiploid PHW-SA [21]. Banding patterns and chromosome morphology allow identification of the homologues of the chromosome of *P. huashanica* in wheat- *P. huashanica* introgression lines. In the current study, based on similarities with the standard C-banded karyotype of common wheat and *P. huashanica* [23], [24], the sharp telomeric bands from *P. huashanica* 3Ns chromosome were distinctly identifyied in lines PW11, PW11-2, PW11-5, and PW11-8 (Figure 1b, f, g, h).

GISH is a powerful and efficient tool for positioning and measuring the alien chromatin integrated into wheat genomes [29], [32]. Kang et al. revealed that the high frequencies of chromosomal breakage and translocation occurring in the amphiploid PHW-SA by use of GISH technique, which may represent a Robertsonian translocations between *P. huashanica* and wheat chromosomes [21]. In the present study, GISH using *P. huashanica* DNA as a probe revealed that the lines PW11-2, PW11-5 and PW11-8 were wheat-*P. huashanica* 3Ns chromosome addition line, 3Ns/1B substitution line and T3BL-3NsS translocation line (Figure 1i, j, k).

SSR markers can also be used to distinguish chromosome substitution lines and translocation lines of wheat [26], [30], [33], [34]. In combination of C-banding, GISH and SSR analyses, the wheat chromosome that was substituted by the 3Ns chromosome of *P. huashanica* in substitution line PW11-5 proved to be chromosome 1B (Figure 1g, j, 5). In translocation line PW11-8, a fragment derived from chromosome 3BL of J-11 was replaced by a fragment of the *P. huashanica* donor (Figure 1h, k, 6). Furthermore, marker *Xgwm161*, which maps proximal to the centromere of chromosome 3D, generated a polymorphic band between J-11 and *P. huashanica*, the amphiploid PHW-SA, PW11-2, PW11-5 and PW11-8 (Figure 4). This indicated that all the lines could carry group 3 chromatin from *P. hushanica*. Therefore, amplification products of this primer pairs could be used to develop the specific marker and used to detect *P. huashanica* chromatin in wheat-*P. huashanica* progenies.

### The wheat–*P. huashanica* introgression lines are new sources of resistance to stripe rust

The occurrence and spread of virulent stripe rust races, such as CYR32 and its variants, poses a serious threat to global wheat production. Few modern cultivars or adapted germplasms possess adequate resistance. In the long term, the major resistance genes have turned out to be non-durable as virulence in the pathogen population was selected or it rapidly evolved following the introduction of such resistance genes [1]. Consequently, a constant search and transfer from novel and effective sources of resistance is necessary to counterbalance the continuous evolution of rust pathogens. So far, More than 70 stripe rust resistance (*Yr*) genes have been officially or provisionally designated in wheat and more than 10 genes of these have been transferred from alien species [5], [6], [35], [36]. Except for *Yr28*, *Yr35* and *Yr36*, all other alien genes have been transferred from nonprogenitor species. Most of the designated *Yr* genes confer a hypersensitive reaction and the majority of these are no longer effective due to evolution of new virulences in the pathogen [37]. *Yr9*, which had been the basis of resistance in the widely grown Chinese cultivars Fan 6 and derivatives, has now become ineffective against a new virulence designated as CYR32 [2]–[4].

Here, we showed that the stripe rust resistance gene(s) present in wheat–*P. huashanica* introgression lines is effective against predominant *Pst* pathotype CYR32, and we characterized the chromosomal segment harbouring the resistance gene in the T3BL-3NsS translocation. Other genes *Yr5*, *Yr10*, *Yr24*, *Yr37*, *Yr40*, *Yr41* and *YrP81* are effective against CYR32, and all of them, except *Yr10*, *Yr41* and *YrP81*, are of alien origin and provide seedling resistance [7], [38]. However, most of these genes, which are derived from wild relatives of wheat, are located on chromosome translocations that include large donor segments that harbour genes possibly deleterious to agronomic and quality traits [7]. In the present study, we successfully produced the translocation line with smaller alien chromosome segments containing the resistance gene. It could be a useful bridge for the transference of the stripe rust resistance gene of *P. huashanica* to common wheat. It is vital that the new sources of resistance and effective resistance genes be identified, characterized, and deployed into adapted germplasm and varieties.

### The SS translocation between wheat and *P. huashanica* chromosomes

Four methods of chromosome engineering are often employed in wheat breeding programs to induce alien translocation, including (I) tissue culture [32], [39]; (II) use of ionizing radiation [27], [40]; (III) exploitation of centric break-fusion of univalent chromosomes [41]; (IV) induction of homologous pairing and crossingover by manipulating the *ph* gene [42]. These methods, however, require a great deal of painstaking cytological work and are very time-consuming, with low frequency of induced alien translocations, a majority of which usually occur between chromosome arms or large segments of wheat and alien species [22], [27]. Most of the gained large alien segments cannot completely compensate for the loss of the corresponding segments of wheat chromosomes in these translocations and/or may contain adverse chromatin along with the desired genes, thus limiting the wider application of the alien translocation lines in wheat improvement [26], [43]. To overcome these shortcomings, the most desirable approach is to induce the small-segment-translocation (SS translocation), i. e. the insertion of a small alien segment into a recipient chromosome without loss of recipient chromatin.

The great value of SS translocation in plant breeding has been recognized for a long time. Ren et al. found that in the selfing generation of monosomic addilion lines, not only the single added rye chromosomes in wheat were eliminated rapidly, but also the wheat chromosomes existing in pairs exhibited a tendency toward cytological instability, leading to occurrence of translocations between different arms of wheat and rye chromosomes with a considerable frequency [43]. Ren and Zhang indicated that the technique which was based on the genetic instability caused by monosomic addition of rye chromosome in wheat was an effective means to induce wheat-rye SS translocation [44]. In our study, because a pure line of wheat was used as the recepient, variation of analyzed plants using the wheat parent as control. Then it was possible to find these wheat plants, which showed variation as compared with their wheat parents. Among the selfed progenies of the monosomic addition line PW11, a SS translocation line PW11-8 were selected, in which the segments of *P. huashanica* 3NsS chromosomes could be decided by C-banding, GISH and SSR, and they exhibited the resistance to stripe rust from *P. huashanica* parent. All these results indicated that the SS translocations could be produced through the method described in this paper, which was similar to the report of the wheat-rye SS translocation by Ren and Zhang [44]. Due to the existence of *kr* genes, wheat can be crossed easily with its distantly-related species. It is not difficult to develop the monosomic addition lines from the progenies of hybrids between wheat and alien species. The monosomic addition lines can be used to induce SS translocation, as an efficient tool for transferring alien genes into wheat.

## Materials and Methods

### Plant materials

*Psathyrostachys huashanica* Keng ex Kuo (2n = 2x = 14, NsNs) accession ZY3157 was collected from Huashan Mountain, Shaanxi, China by Profs. Yen C and Yang JL (Sichuan Agricultural University). J-11 (*Triticum aestivum* L., 2n = 6x = 42, AABBDD) was a Sichuan white grained cultivar. A new amphiploid between J-11 and *P. huashanica*, designated PHW-SA (2n = 8x = 56, AABBDDNsNs), with resistance to wheat stripe rust disease, was obtained [15]. The parental wheat cultivar J-11 and *P. huashanica* were included in the present study in assessment of resistance to stripe rust and molecular cytogenetic analysis as controls. Wheat cv. Mingxian 169 was used as a susceptible control for stripe rust disease response tests. Chinese Spring (CS) wheat was used as a source of blocking DNA in GISH analysis. To identify and locate markers specific for wheat and *P. huashanica* chromosome of the wheat- *P. huashanica* introgression lines, the eight CS nulli-tetrasomic (NT) lines N1AT1B, N1BT1A, N1BT1D, N1DT1B, N3AT3B, N3BT3A, N3BT3D and N3DT3B were used. The NT lines were kindly provided by Dr. Bikram S. Gill, Department of Plant Pathology, Kansas State University, Manhattan, KS, USA. Seeds of the above mentioned materials are maintained at Triticeae Research Institute, Sichuan Agricultural University, Sichuan, China (SAUTI).

### Ethics statement

The below observational and field studies were approved by the Wildlife Conservation and Nature Reserve Management Office of Shaanxi Province, China, and was also permitted by Huashan Mountain national park, Shaanxi (06-1357). The study was carried out in strict accordance with the Laboratory System of Sichuan Agricultural University.

### Meiosis analyses

For meiotic studies, chromosome paring at metaphase I (MI) of pollen mother cells (PMCs) was observed as previously reported by Kang et al. [16]. Briefly, the young spikes of wheat at the appropriate stages were fixed in Carnoy's fixative I (3 parts of 95% ethanol: 1 part of glacial acetic acid) for 24 h and then stored in 70% ethanol. The slides of PMCs were made in 1% iron acetocarmine. Chiasmata frequency was estimated from the number of chromosome arm paired per cell at MI. Cytological observations and documentation were made with an Olympus BX-61 microscope coupled with a Photometrics SenSys CCD camera.

### Giemsa C-banding

The Giemsa C-banding technique followed the procedure of Gill et al. with slight modifications [23]. Seeds were germinated in petri dishes on moist filter paper. Root tips 1–3 cm long were pretreated in ice water at 0–4°C for 24 h. Then, the root tips were fixed in Carnoy's fixative I (3 parts of ethanol: 1 part of glacial acetic acid) for 24 h at room temperature. Root tips were squashed in 45% acetic acid. The cover slip was removed by freezing in liquid nitrogen and the slides were treated with 99% ethanol at room temperature overnight and then air dried for a few minutes. Next, the preparations were incubated in 0.2 M HCl at 60°C for 2.5 min, washed in distilled water for a few minutes, incubated in saturated barium hydroxide solution at room temperature for 7–8 min, rinsed in hot water at 23–24°C for 2 min and then in distilled water for 30 min, incubated in 2×SSC at 60°C for 1 h. The slides were directly transferred to 4% Giemsa staining solution (Fisher) in phosphate buffer for up to 20 min. The staining of chromosomes was monitored until optimal staining was observed. The identification of Ns chromosomes of *P. huashanica* followed the report from Wang et al. and Kang et al. [21], [24].

### Genomic *in situ* hybridization (GISH)

Root tips from germinating seeds were pretreated in ice-cold water for 24–28 h, fixed in ethanol∶acetic acid (3∶1 v/v) for 24 h at room temperature and then stored in a refrigerator. Each root tip was squashed in a drop of 45% acetic acid and frozen in a refrigerator at −76°C. Total genomic DNA of *P. huashanica* and CS were extracted from fresh leaves by the CTAB (cetyltri methylammonium bromide) method [45].

GISH analysis was performed according to Reader et al. with minor modifications [46]. *P. huashanica* DNA was labelled with digoxigenin-11- dUTP by a nick translation mix (Roche, Mannheim, Germany) then used as the hybridization probe in order to detect *P. huashanica* chromatin. Unlabelled CS DNA was sheared by autoclaving to 200–400 bp pieces at 120°C for two minutes and used as a competitor at 50 times the quantity of the probe amount in order to block common sequences in the hybridization step. 50 *µl* of denatured hybridization solution containing 2×SSC, 10% dextran sulphate, 0.2% sodium dodecyl sulphate, 1 ng/µl labelled probe DNA together with the competitor DNA, were loaded per slide and incubated for 2 h at 65°C. Post- hybridization washes were in 2×SSC at room temperature for 5 min, in 2×SSC at 42°C for 10 min, 2×SSC at room temperature for 5 min. Chromosomes were counterstained with propidium iodide (PI) (1 *µ*g/ml). Images were captured with a cooled CCD camera (Photometrics CoolSNAP fx: Roper Scientific) using a fluorescence microscope (Olympus BX61). To construct a generalized karyotype, 3 images from two slides after GISH treatments were analyzed.

### SSR analysis

Genomic DNA was isolated from the wheat- *P. huashanica* introgression lines and the two parents as previously described [45]. One hundred and eighty three simple sequence repeats (SSR) primer pairs [47], [48] were used to characterize the genomic composition of the wheat-*P. huashanica* lines. These primers amplify clear and repeatable products from the wheat lines and are distributed uniformly throughout all of the wheat chromosomes. Twenty microlitres of each reaction mixture contained 10 mM of Tris–HCl (pH 8.3), 50 mM of KCl, 3.0 mM of MgCl_2_, 5.0 mM of dNTP each, 5.0 mM of primers each, 60 ng of genomic DNA and 1 unit of *Taq* polymerase. DNA amplification was performed in a GeneAmp 9700 Thermal Cycler (Applied Biosystems Inc., California, USA), which was programmed for 5 min at 94°C; 35 cycles of 1 min at 94°C, 1 min at 50–60°C, and 1 min at 72°C; and 10 min at 72°C for a final extension. The PCR products were separated on 6% polyacrylamide denaturing gels and were visualized following silver-staining.

### Screening for stripe rust response

The wheat- *P. huashanica* introgression lines were grown in a growth chamber. Seedlings were inoculated at the three-leaf stage with the widely virulent *Puccinia striiformis tritici* (*Pst*) race CYR32 for evaluating their reactions according to previously described methods [49]. Seedlings were misted through an inlet until sufficient dew was formed then incubated for 12 h in darkness at 14°C followed by a 24 h light period at the same temperature and subsequent growth under natural daylight at 16–18°C. Seedling infection types (IT) were evaluated 3–4 weeks after inoculation when the pustules on susceptible check Mingxian 169 was fully developed. Infection types were classified on a 0–4 rating scale: 0 = immunity, no visible symptoms, 0;  = necrotic flecks without uredinia, 1 = small uredinia surrounded by distinct necrosis, 2 = small to medium- sized uredinia with chlorosis and necrosis, 3 = moderate-sized sporulating uredinia surrounded by chlorosis, and 4 = abundant sporulation without chlorosis or necrosis [50].
